# Analysis on the Effect of Radiofrequency Ablation and Electrocautery in the Treatment of Vaginal Intraepithelial Neoplasia

**DOI:** 10.1155/2023/9432073

**Published:** 2023-02-06

**Authors:** Jia-Wei Xiong, Zhi-Xin Lin, Liu He, Yan-Hua Zhang, Gui-Chan Zeng, Qing-Hua Gui

**Affiliations:** Gynecology and Cervical Center of Xiangzhu Section of the Maternal and Child Health Hospital of Guangxi Zhuang Autonomous Region, Nanning 530002, China

## Abstract

**Objective:**

This research intends to investigate the clinical efficacy of radiofrequency ablation and electrocautery in treating grade I or II vaginal intraepithelial neoplasia (VaIN).

**Methods:**

This is a single-center retrospective study, which collected the clinical data of 100 patients with VaIN diagnosed by colposcopy and pathological biopsy in the Gynecology and Cervical Center of Xiangzhu Branch of the Maternal and Child Health Hospital of Guangxi Zhuang Autonomous Region between January 2020 and June 2021. Patients were divided into the study group (radiofrequency ablation treatment) and the control group (electrocautery) according to differences in treatment approaches. 6- and 12-month follow-ups were performed on all patients. Gynecological examination results, liquid-based thin-layer cytology (TCT), negative conversion of human papillomavirus (HPV), curative effects, and prognosis were recorded.

**Results:**

All patients completed regular follow-ups that lasted for 6 and 12 months. The 6- and 12-month cure rates of the study group were 76.0% and 92.0%, respectively, and the data in the control group were 70.0% and 82.0%, respectively. In terms of the 6- and 12-month negative conversion rates of HPV, the data in the study group were 68.0% and 78.0%, versus 60% and 68% in the control group, respectively. The lesion duration rate showed no statistical significance between the study group (8.0%) and the control group (*P* > 0.05). The analysis of postoperative follow-up complications revealed that the study group had a statistically lower overall incidence of vaginal bleeding, excessive vaginal discharge, vaginal burning sensation, and decreased vaginal elasticity than the control group (8.0% vs. 24.0% *P* < 0.05).

**Conclusion:**

Both radiofrequency ablation and electrocautery have obvious clinical effects in patients with grade I or II VaIN, but the former contributed to fewer operative complications and a good prognosis, which deserves clinical promotion.

## 1. Introduction

Vaginal intraepithelial neoplasia (VaIN) refers to atypical proliferative changes of varying degrees confined to the vaginal squamous epithelium, of which high-grade VaIN is a precancerous lesion with unclear pathogenesis that it is reported to be related to cervical intraepithelial neoplasia (CIN) and high-risk (hr) human papillomavirus (HPV) infection [1, 2]. VaIN, which bears responsibility for approximately 0.4% of lower genital tract intraepithelial lesions used to be rare clinically, but the incidence has been increasing in recent years [3]. Most patients with VaIN are detected during cervical screening and colposcopy, as the majority of them have no obvious symptoms except increased vaginal secretion or a small amount of bleeding after sexual intercourse in few cases, as well as the particularity and concealment of vaginal anatomy [4, 5]. Given few clinical studies on VaIN at home and abroad, the management of the disease deserves more clinical attention. As we all know, the general treatment of VaIN includes drug therapy, physiotherapy, and surgical treatment, with no recognized best method at present [6]. There are various treatments for VaIN, including follow-up without treatment, local medication, laser treatment, radiotherapy, lesion resection, and vaginal resection [7, 8]. Surgical methods include local resection, CO_2_ laser ablation, CO_2_ laser skin colectomy, and laparoscopic upper vaginal resection. Medical management is based on topical medications in the vagina, such as 5% imiquimod cream, 5-fluorouracil cream, and local estrogen. Intracavitary radiotherapy includes low- and high-dose rate brachytherapy [9].

Radiofrequency ablation is a minimally invasive technique to eliminate pathological changes by generating heat from high-frequency radio frequency waves, which leads to cell coagulation and necrosis [10]. Electrocautery is performed by direct electrocautery of the diseased tissue with a high-frequency electrotome [11]. In recent years, there is little information about VaIN treatment by the two methods. Most patients develop complications such as decrustation, and bleeding, infection, and tissue adhesion after physiotherapy [9]. Therefore, to better treat VaIN and reduce complications, patients with grades I-II VaIN were treated with radiofrequency ablation and electrocautery, combined with Baofukang suppositories after operation, in order to explore the clinical effects of the two treatments.

## 2. Material and Methods

### 2.1. Study Participants

The clinical data of 100 patients with VaIN diagnosed by colposcopy and pathological biopsy admitted between January 2020 and June 2021 to the Gynecology and Cervical Center of Xiangzhu Branch of the Maternal and Child Health Hospital of Guangxi Zhuang Autonomous Region were collected for retrospective analysis. This study was conducted after being approved by the Hospital Ethics Committee, and all participants provided informed consent. Inclusion criteria: the diagnosis of VaIN by thin-layer cytology (TCT), hrHPV testing (Roche Cobas 4800), colposcopy, and histopathology. Exclusion criteria: pregnant and breast-feeding patients; patients with cervical cancer or other malignancies of the reproductive system; patients with acute reproductive tract infection; patients with other systemic diseases, severe immune disease, gonorrhea, or acute pelvic inflammatory disease; patients with related surgical contraindications. According to the treatment method, 50 patients with radiofrequency ablation treatment were included in the study group, and 50 with electrocautery therapy were included in the control group. The clinical data (age, HPV subtype, TCT results, histological grade, cervical involvement, operation process, prognosis, etc.) of all patients were collected.

### 2.2. Treatment and Follow-Up

#### 2.2.1. Electrocautery

The patients in the control group were treated with electrocautery; the procedure was performed 3 to 7 days postmenstruation. After the bladder was emptied, the subject was put in the bladder lithotomy position, the vulva was disinfected, and the cervix was exposed with a vaginal speculum. The location and extent of vaginal wall lesions were determined by a 3–5% acetic acid test and Lugol's iodine solution test [12] under colposcopy. The electrode plate was placed in the lumbosacral area of the patient, and the lesion surface was treated with high-frequency electrocautery. The operation ended until the surface of the tissue was covered by a yellow and coagulated layer without bleeding. Postoperatively, Baofukang suppositories (2 tablets a day) were used in combination with local treatment, which were placed deep in the vagina, for 2 weeks. Re-examination was performed 6 months after the operation.

#### 2.2.2. Radiofrequency Ablation

The patients in the study group were treated with radiofrequency ablation; the procedure was also performed 3 to 7 days after menstruation. Similarly, the bladder was emptied preoperatively, and the subject was placed in the bladder lithotomy position. Following vulva disinfection, the cervix was exposed with a vaginal speculum. The location and range of vaginal wall lesions were determined by 3%–5% acetic acid test and the Lugol's iodine solution test under colposcopy. The rated power of radio frequency output was set to 30 W, and the electrode plate of radio frequency therapeutic instrument was placed in the lumbosacral area of the patient. The head of the self-coagulating knife was close to the focus surface, so that the lesion surface could be completely solidified and turned white. After the operation, Baofukang suppositories were applied in combination with local treatment, which were placed deep in the vagina, 2 tablets a day for 2 weeks. Patients were reexamined 6 months postoperatively.

#### 2.2.3. Follow-Up and Efficacy Evaluation

The postoperative curative effects of the two groups were compared. Since 6- and 12-month follow-ups were performed on all patients. Gynecological examination, TCT, HPV, and colposcopy were performed during the follow-up. Patients with abnormalities in the former tests were further examined by colposcopy. Cure: normal TCT results after treatment, along with negative conversion of HPV, no obvious vinegar white epithelium as indicated by colposcopy, and uniform staining shown by the iodine test; improvement: normal or abnormal TCT results after treatment, with or without negative conversion of HPV, focal thin vinegar white epithelium by colposcopy, uneven staining shown by the iodine test, and decreased VaIN grading by biopsy. Persistence or progression: TCT abnormalities were reexamined after treatment, with or without negative conversion of HPV, thin vinegar white epithelium or focal thick vinegar white epithelium by colposcopy, nonstaining by the iodine test, and no decrease in VaIN grading by colposcopy-guided biopsy.

#### 2.2.4. Statistical Analysis

The data were collected statistically processed by SPSS25.0 statistical software (SPSS, Inc, Chicago, USA). The two independent samples *t* test was used for between-group comparisons of measurement data represented by ($\bar{\chi }$ ± *s*). The between-group comparison of counting data represented by percentage [*n* (%)] was the *χ*^2^ test. Statistical significance was indicated by a *P* value <0.05.

## 3. Results

### 3.1. Clinical Characteristic of Participants

The two groups showed similar clinical characteristics (Table 1). Patients in the study group aged 23–68 (mean: 44.84 ± 12.65), versus 23–62 years old (mean: 42.92 ± 11.08) in the control group, showing no statistical significance (*P* > 0.05). Among the 50 patients in the study group, 42 patients were diagnosed as stage I VaIN, including 39 with hrHPV positive and 30 patients with I-II CIN; the results of TCT showed 13 cases of NILM, 12 cases of ASCUS, 20 cases of LSIL, and 5 cases of HSIL. While in the control group, 34 patients were HPV positive and 28 patients were complicated with I-II CIN; the results of TCT showed NILM, ASCUS, LSIL, and HSIL in 15, 15, 16, and 4 cases, respectively.

### 3.2. Follow-Up and Prognosis

At 6-month follow-up, 38 of the 50 patients in the study group were cured, showing no obvious abnormality in HPV and TCT detection; 9 patients improved, showing normal or abnormal TCT reexamination results, focal thin vinegar white epithelium by colposcopy, uneven staining by the iodine test, and decreased VaIN grading by biopsy, with or without negative conversion of HPV; 3 patients showed disease progression, exhibiting abnormal TCT results, thin vinegar white epithelium or focal thick vinegar white epithelium by colposcopy, nonstaining by the iodine test, and no decrease in VaIN grading by colposcopy biopsy, with or without negative conversion of HPV. Among the 50 patients in the control group, 35 patients were cured, 8 patients improved, and 7 patients persisted or progressed. During the follow-up to 12 months, 46 of the 50 patients in the study group were cured, while 4 patients showed disease progression; 41 of the 50 patients in the control group were cured, and 9 patients continued or progressed. The study group had a higher cure rate and a lower disease persistence rate than the control group, but without statistical significance (*P* > 0.05) (Table 2).

### 3.3. Clearance Rate of HPV Infection

The 6-month follow-up revealed a negative conversion rate of HPV of 68% in the study group and 60% in the control group. While the negative conversion rate at 12-month follow-up was 78% in the study group, versus 68% in the control group. The data determined higher but nonsignificantly different negative conversion rates of HPV in the study group than the control group at 6 and 12 months (*P* > 0.05) (Table 3).

### 3.4. Adverse Reactions

**Figure 1:**
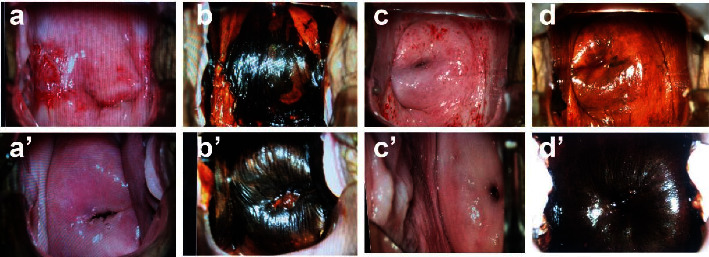
Comparison of pre and posttreatment colposcopy findings between two groups (magnification × 7.5, integrated photoelectric system, Leisegang, Germany). Preoperative electrocautery (a, b) showed local thick vinegar white epithelium by colposcopy as well as uneven staining by the iodine test; thin vinegar white epithelium was still observed by postoperative colposcopy, and the iodine test showed was slightly uneven staining (a', b'). Preoperative radiofrequency ablation (c, d) indicated local thick vinegar white epithelium by colposcopy and uneven staining by the iodine test; the postoperative colposcopy did not show obvious vinegar white epithelium, and the iodine test showed uniform staining (c', d').

During the follow-up, the vaginal mucosa at the operation site examined by colposcopy in the radiofrequency ablation group was smooth and elastic, without obvious scarring and contracture, and no contact bleeding; only 4 patients had a small amount of vaginal bleeding and more vaginal discharge after operation, which were relieved spontaneously after Baofukang suppository treatment. In the electrocautery group, there were 10 patients with slight scarring and contracture, a small amount of vaginal bleeding, a slight burning sensation, and more vaginal discharge, and these symptoms were relieved by themselves after treatment with Baofukang suppositories. The incidence of postoperative complications was 8.0% (4/50) in the radiofrequency ablation group and 24.0% (12/50) in the electrocautery group, showing no significant difference (*P* < 0.05) (Table 4). At the end of follow-up, the vaginal anatomical structure of all patients remained unchanged. As presented in Figure 1, before electrocautery, local thick vinegar white epithelium was seen by colposcopy, with uneven staining as indicated by the iodine test; postoperatively, colposcopy still showed thin vinegar white epithelium, and the iodine test revealed slightly uneven staining. Before radiofrequency ablation, colposcopy suggested local thick vinegar white epithelium, and the iodine test showed uneven staining; the postoperative colposcopy did not show obvious vinegar white epithelium, and the iodine test suggested uniform staining.

## 4. Discussion

VaIN is a precancerous lesion composed of a typical hyperplastic squamous cell groups, including atypical hyperplasia and carcinoma in situ, which can be classified as grades I-III VaIN according to the degree of atypical hyperplasia. Studies have reported that low-grade VAIN has a tendency to self-heal, but not all can be reversed with some of the lesions persist or even progress [13]. Risk factors for VaIN include hrHPV infection, CIN, cervical cancer, and hysterectomy [14]. It is reported that most VaIN patients are complicated with I-II CIN [15]. In this study, 58 patients were found to be complicated by I-II CIN. HPV can cause cervical and vaginal infections. VaIN may be the continuation of CIN, and different types of HPV infection may cause vaginal lesions. Therefore, VaIN diagnosis and treatment should be given equal importance premised on the diagnosis and management of cervical lesions. It is reported that almost all VaIN patients have been infected with HPV [16], and hrHPV infection is the main cause of VaIN. However, there are also HPV-negative VaIN patients, and most of them are diagnosed by colposcopy because of abnormal TCT results. In this study, 73 cases were positive for hrHPV and 27 cases were negative, with a positive rate of hrHPV of 73%. hrHPV infection is an important factor affecting the progression, recurrence, and prognosis of VaIN [17]. Thus, we should attach special importance to HPV infection in the diagnosis, treatment, and follow-up of VaIN patients. Age is also shown to be an important risk factor for VaIN [18]: postmenopausal women are more susceptible to VaIN than premenopausal women, mainly due to the lack of estrogen in the former, resulting in thin vaginal epithelium, decreased local vaginal resistance, and increased possibility of hrHPV infection [19]. In this study, the oldest patient was 68 years old, and 47.0% of the patients aged over 45. It can be seen that older women are more likely to develop VaIN. It is worth noting that 3% of the participants aged under 25. Studies have shown that the early age of first sexual behavior is also associated with a higher risk of developing HPV infection and precancerous lesions [20]. Therefore, clinical attention should also be paid to young patients, and the application of early TCT and HPV screening increases the detection rate of the disease in young women.

The current treatment of VaIN is roughly the same as CIN, with fewer adverse reactions, which is suitable for young and mild patients; however, the long treatment course leads to poor patient compliance, resulting in low stability of treatment efficacy, while surgical treatment is traumatic and requires high requirements for operators, with postoperative complications that have a certain impact on the quality of life of patients. Therefore, choosing an appropriate treatment is of utmost importance. In recent years, a number of studies have reported better clinical effects of CO_2_ laser ablation and photodynamic therapy in the treatment of VaIN. For example, Han et al. [21] used photodynamic therapy to treat VaIN, and found that the cumulative cure rate was 87.5% after 6 months, but with the presence of clinical symptoms such as postoperative lower abdominal pain, increased vaginal discharge, and local pruritus. Luyten et al. [22] reported a cure rate of 87.0% when using CO_2_ laser to treat VaIN patients. Most patients need repeated laser treatment. For instance, CO_2_ laser ablation has a certain residual rate, which increases the possibility of repeated operations, resulting in an increased risk of postoperative vaginal stricture, shortening, scar formation, and difficulty in sexual intercourse, as well as the need for long-term follow-up. It can be seen that each treatment has its advantages and disadvantages. In the control group, electrocautery was performed to scald the vaginal lesions, and the thermal effect was used to carbonize the tissue protein and occlude the blood vessels; thus, inducing the atrophy and even necrosis and shedding of the diseased wound tissue. Moreover, due to the mild and uniform heating during electrocautery, the action point is relatively accurate, and the depth and treatment can be judged according to the changes in the color of the wound surface, with high safety. It has been reported that the cure rate of electrocautery for VaIN is 25%–88% [23]. In this study, 50 patients with VaIN were treated with electrocautery, a procedure with the advantages of simple operation and fast recovery. The cure rates in 6 and 12 months were found to be 70% and 82%, respectively, which is consistent with previous studies. The study group adopted radiofrequency ablation. Compared with other physiotherapy, radiofrequency ablation performed colposcopically with the help of acetic acid white test and iodine test can more accurately determine the location and extent of the lesions and destroy the lesions accurately and efficiently without forming coking toxic components. The principle of radiofrequency ablation lies in the use of radiofrequency current to flow through human tissue. The positive and negative ions in cells move rapidly under the action of electromagnetic fields. When friction occurs between cells and molecules, the temperature of the lesion site increases, and the evaporation of water inside and outside the cell accelerates, which effectively promotes the drying and pyknosis of cells, allowing for elimination of the diseased tissue and effective clearance of HPV infection [7]. In this study, we applied radiofrequency ablation to treat VaIN patients for the first time. This method has the advantages of simple operation, short operation time, almost no intraoperative bleeding, and high safety. The cure rate was 76% in 6 months and 92% in 12 months, which was higher compared with the electrocautery group.

In addition, the negative conversion rates of HPV in the radiofrequency ablation group and electrocautery group in 6 months were 68% and 60%, respectively, and those in 12 months were 78% and 68%, respectively, showing no statistical significance (*P* > 0.05). The increase in the negative conversion rate of HPV during the follow-up period also indicates the long-term effect of radiofrequency ablation. While resolving the lesion, the treatment can lower the viral load to some certain extent and even eliminate HPV infection. The results showed that the negative conversion rate of other hrHPV was higher than that of HPV16/18.

In order to better treat VaIN and reduce the occurrence of complications after physiotherapy, all patients with VaIN were treated with Baofukang suppositories postoperatively. As a traditional Chinese medicine suppository, the Baofukang suppository is mainly composed of zedoary turmeric oil and borneol. Zedoary turmeric can activate blood to remove blood stasis, relieve pain, and remove saprophytic muscle; while borneol has the effects of inducing resuscitation, alleviating swelling and pain, removing saprophytic muscles, cooling blood, and relieving itching [24]. The application of Baofukang suppositories to the wound after radiofrequency ablation and electrocautery can not only accelerate the renewal and repair of injured tissue, inhibit abnormal tissue proliferation, promote wound scabbing and decrustation but also block virus reproduction and enhance the ability of human self-immunity to resist and eliminate HPV infection.

Through the analysis of the curative effect of radiofrequency ablation in patients with VaIN, this study found that radiofrequency ablation was effective in patients with stages I-II VaIN, which could not only accurately locate the target tissue of injury but also reduce the damage to the surrounding normal tissue. Postoperative treatment with Baofukang suppositories is conducive to accelerating wound healing, alleviating vaginal scar hyperplasia and tissue adhesion, and reducing the symptoms of postoperative vaginal bleeding and vaginal discharge. The vaginal anatomy and function remained good after treatment, but a small number of patients in the electrocautery group developed complications such as vaginal burning sensation, decreased vaginal elasticity, and stricture, which affect their quality of life and their satisfaction with subsequent colposcopy.

## 5. Conclusion

To sum up, both radiofrequency ablation and electrocautery in combination with Baofukang suppositories are effective in the treatment of VaIN, which can promote lesion healing, reduce recurrence, and improve the cure rate of patients. But relatively speaking, radiofrequency ablation has higher safety, which contributes to fewer postoperative complications and a higher cure rate. Thus, radiofrequency ablation combined with Baofukang suppositories can be given priority in the treatment of stages I-II VaIN patients. However, there is still some room for improvement in this study. The sample size of this study may be too small to detect a difference between the two approaches. Consequently, a multicenter, large sample, and long-term follow-up study is needed to confirm the research findings.

## Figures and Tables

**Table 1:** Baseline characteristics of the patients.

Characteristics	Study group (*n* = 50)	Control group (*n* = 50)	*χ* ^2^/*t* value	*P* value
Age *n* (%)			0.807	0.421
≤45	23 (46.0)	30 (60.0)		
>45	27 (54.0)	20 (40.0)		
Mean age	44.84 ± 12.65	42.92 ± 11.08		

VaIN diagnosis			0.332	0.564
VaIN I	42 (84.0)	44 (88.0)		
VaIN II	8 (16.0)	6 (12.0)		

HPV subtype			1.275	0.529
Negative	11 (22.0)	16 (32.0)		
Single HPV16/18	10 (20.0)	9 (18.0)		
Other hrHPV	29 (58.0)	25 (50.0)		

TCT results			1.032	0.794
NILM	13 (26.0)	15 (30.0)		
ASCUS	12 (24.0)	15 (30.0)		
LSIL	20 (40.0)	16 (32.0)		
HSIL	5 (10.0)	4 (8.0)		

Cervical lesion involvement			0.371	0.831
No	20 (40.0)	22 (44.0)		
CIN I	23 (46.0)	20 (40.0)		
CIN II	7 (14.0)	8 (16.0)		

VaIN: vaginal intraepithelial neoplasia; HPV: human papillomavirus; TCT: thin-layer cytology; NILM: negative for intraepithelial lesion or malignancy; ASCUS: atypical squamous cell of undetermined significance; LSIL: low-grade squamous intraepithelial lesion; HSIL: high-grade squamous intraepithelial lesion; CIN: cervical intraepithelial neoplasia.

**Table 2:** Comparison of clinical efficacy between the two groups after treatment.

Group	*n*	6-month follow-up			12-month follow-up		
		Cure	Improvement	Persistence or progression	Cure	Improvement	Persistence or progression
Study group	50	38 (76.0)	9 (18.0)	3 (6.0)	46 (92.0)	0 (0)	4 (8.0)
Control group	50	35 (70.0)	8 (16.0)	7 (14.0)	41 (82.0)	0 (0)	9 (18.0)
*χ* ^2^ value		0.457	0.071	1.778	2.210	—	2.210
*P* value		0.499	0.790	0.182	0.137	—	0.137

**Table 3:** Comparison of posttreatment HPV-negative conversion.

Group	*n*	Negative conversion of HPV after 6 months of follow-up		Negative conversion of HPV after 12 months of follow-up	
		*n*	%	*n*	%
Study group	50	34	68.0	39	78.0
Control group	50	30	60.0	34	68.0
*χ* ^2^ value		0.694		1.268	
*P* value		0.405		0.260	

**Table 4:** Comparison of postoperative complications.

Group	*n*	Vaginal bleeding		Vaginal discharge		Vaginal burning sensation and decreased elasticity		Total number of complications	
		*n*	%	*n*	%	*n*	%	*n*	%
Study group	50	2	4.0	2	4.0	0	0	4	8.0
Control group	50	3	6.0	4	8.0	5	10.0	12	24.0
*χ* ^2^ value		0.211		0.709		5.263		4.762	
*P* value		0.646		0.400		0.022		0.029	

## Data Availability

The labeled dataset used to support the findings of this study are available from the corresponding author upon request.

## References

[1] Lamos C., Mihaljevic C., Aulmann S. (2016). Detection of human papillomavirus infection in patients with vaginal intraepithelial neoplasia. *PLoS One*.

[2] Li Z., Barron S., Hong W., Karunamurthy A., Zhao C. (2013). Surveillance for recurrent cancers and vaginal epithelial lesions in patients with invasive cervical cancer after hysterectomy: are vaginal cytology and high-risk human papillomavirus testing useful?. *American Journal of Clinical Pathology*.

[3] Gurumurthy M., Cruickshank M. E. (2012). Management of vaginal intraepithelial neoplasia. *Journal of Lower Genital Tract Disease*.

[4] Boonlikit S., Noinual N. (2010). Vaginal intraepithelial neoplasia: a retrospective analysis of clinical features and colpohistology. *Journal of Obstetrics and Gynaecology Research*.

[5] Dodge J. A., Eltabbakh G. H., Mount S. L., Walker R. P., Morgan A. (2001). Clinical features and risk of recurrence among patients with vaginal intraepithelial neoplasia. *Gynecologic Oncology*.

[6] Rome R. M., England P. G. (2000). Management of vaginal intraepithelial neoplasia: a series of 132 cases with long‐term follow‐up. *International Journal of Gynecological Cancer*.

[7] Sopracordevole F., Clemente N., Di Giuseppe J. (2020). Clinical characteristics and long-term follow-up of patients treated for high-grade vaginal intraepithelial neoplasia: results from a 20-year survey in Italy. *Journal of Lower Genital Tract Disease*.

[8] Zhang T., Hu R., Tang Y. (2022). The effect of local photodynamic therapy with 5-aminolevulinic acid in the treatment of vaginal intraepithelial lesions with high-risk hpv infection. *Photodiagnosis and Photodynamic Therapy*.

[9] Rountis A., Pergialiotis V., Tsetsa P., Rodolakis A., Haidopoulos D. (2020). Management options for vaginal intraepithelial neoplasia. *International Journal of Clinical Practice*.

[10] Habibi M., Berger R. D., Calkins H. (2021). Radiofrequency ablation: technological trends, challenges, and opportunities. *EP Europace*.

[11] Prussin A. J., Babajanian E., Error M. (2021). Radiofrequency ablation vs electrocautery blinded randomized trial: impact on clinically meaningful outcomes. *Otolaryngology-Head and Neck Surgery*.

[12] Lambert R., Rey J. F., Sankaranarayanan R. (2003). Magnification and chromoscopy with the acetic acid test. *Endoscopy*.

[13] Zhang J., Chang X., Qi Y., Zhang Y., Zhang S. (2016). A retrospective study of 152 women with vaginal intraepithelial neoplasia. *International Journal of Gynecology and Obstetrics*.

[14] Yu D., Qu P., Liu M. (2021). Clinical presentation, treatment, and outcomes associated with vaginal intraepithelial neoplasia: a retrospective study of 118 patients. *Journal of Obstetrics and Gynaecology Research*.

[15] He Y., Wu Y., Zhao Q. (2015). Clinical analysis of patients underwent hysterectomy for stage i cervical cancer or high grade cervical intraepithelial neoplasia with vaginal intraepithelial neoplasia. *Zhonghua Fu Chan Ke Za Zhi*.

[16] Jentschke M., Hoffmeister V., Soergel P., Hillemanns P. (2016). Clinical presentation, treatment and outcome of vaginal intraepithelial neoplasia. *Archives of Gynecology and Obstetrics*.

[17] Darragh T. M., Colgan T. J., Thomas Cox J. (2013). The lower anogenital squamous terminology standardization project for hpv-associated lesions: background and consensus recommendations from the college of american pathologists and the american society for colposcopy and cervical pathology. *International Journal of Gynecological Pathology*.

[18] Song Y., Sui L., Wang Q., Guo Q., Gao S. (2018). Retrospective analysis of liquid based cytology and hpv detection in 1467 cases of vaginal intraepithelial neoplasia. *Journal of Fudan University.(Medical Science)*.

[19] Carcopino X. (2012). I059 epidemiology and risk factors of vaginal intraepithelial neoplasia. *International Journal of Gynecology and Obstetrics*.

[20] Yang W., Gou X., Xu T., Yi X., Jiang M. Cervical cancer risk prediction model and analysis of risk factors based on machine learning.

[21] Han Q., Wu Z., Guo H., Zhang X. (2022). Efficacy and safety of photodynamic therapy mediatied by 5-aminolevulinic acid for the treatment of vaginal high-grade intraepithelial lesions. *Photodiagnosis and Photodynamic Therapy*.

[22] Luyten A., Hastor H., Vasileva T., Zander M., Petry K. U. (2014). Laser-skinning colpectomy for extended vaginal intraepithelial neoplasia and microinvasive cancer. *Gynecologic Oncology*.

[23] Chen L., Hu D., Xu S. (2016). Clinical features, treatment and outcomes of vaginal intraepithelial neoplasia in a Chinese tertiary centre. *Irish Journal of Medical Science*.

[24] Li T., Niu X., Zhang X., Wang S., Liu Z. (2016). Baofukang suppository promotes the repair of vaginal epithelial cells in response to candida albicans. *AMB Express*.

